# Supplementary material for: Molecular Characterization of ZosmaNRT2, the Putative Sodium Dependent High-Affinity Nitrate Transporter of Zostera marina L
Source: Int J Mol Sci. 2019 Jul 26;20(15):3650. doi: 10.3390/ijms20153650 (PMC6695921; doi:10.3390/ijms20153650)
Supplement: Supplementary file 1 [file ijms-20-03650-s001.pdf]

Supplementary material

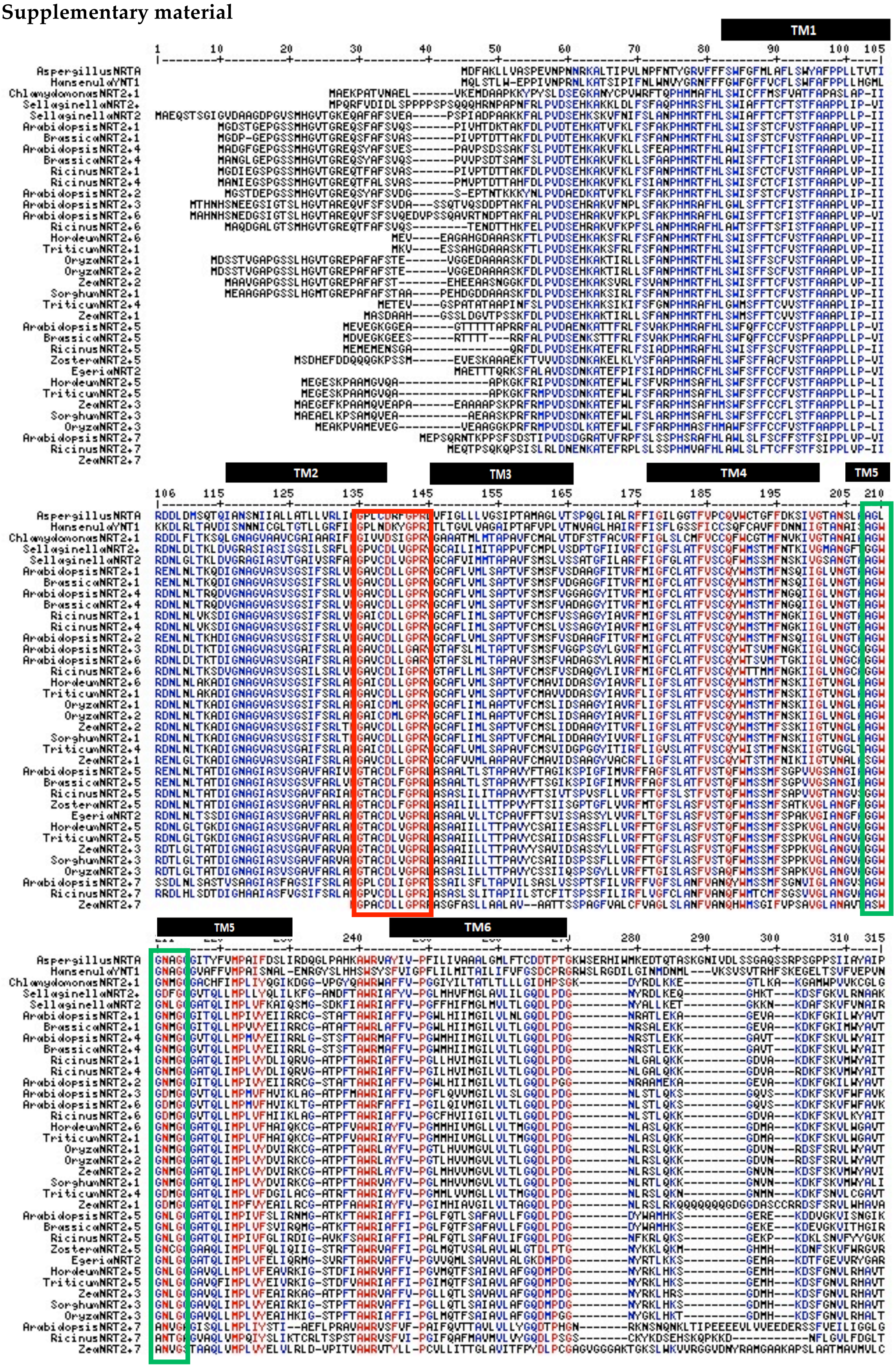

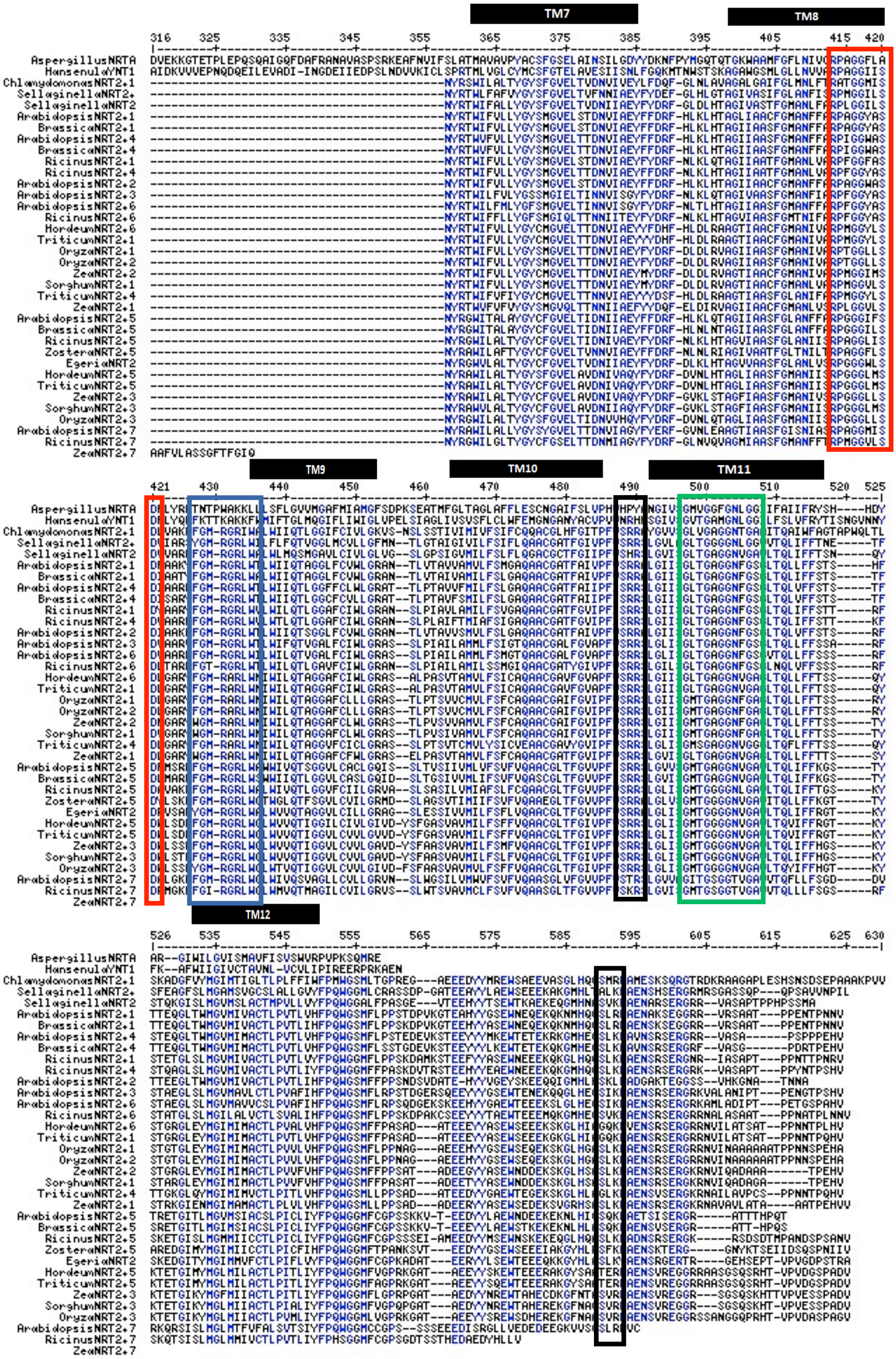
TM7
TM8
TM9
TM10
TM11
TM12

Figure S1. Multiple alignment of NNP family transporters. Sequence alignment was obtained using the MultAlin alignment tool [44]. Red and blue amino acids indicate highly conserved residues, higher than 90% and 50%, respectively. Transmembrane domains of ZosmaNRT2 are overlined and numbered above the sequences. The conserved amino acids for Mayor Facilitator Superfamily motifs (MFS I and II) are framing in red, green for Nitrate Nitrite Porter motifs (NNP I and II), blue for the Photosynthetic NRT2 motif and blak for phosphorylation sites of S/T-X-R/K motifs. The accession numbers of each protein in the NCBI used for the analysis were: *Zostera marina* (NRT2.1, KMZ59016); *Arabidopsis thaliana* (NRT2.1, O82811; NRT2.2, Q9LMZ9; NRT2.3, AED97376; NRT2.4, Q9FJH8; NRT2.5, Q9LPV5; NRT2.6, Q9LXH0; NRT2.7, Q9LYK2); *Brassica napus* (NRT2.1, XP_013729508; NRT2.4, XP_013665864; NRT2.5, XP_013657329) *Ricinus communis* (NRT2.1, XP_002523687; NRT2.4, XP_002523688; NRT2.5, XP_002527899; NRT2.6, XP_002523689; NRT2.7, XP_002524664); *Oryza sativa* (NRT2.2, XP_015623596; NRT2.3, XP_015628524); *Sorghum bicolor* (NRT2.1, XP_002453159; NRT2.3, XP_002456219); *Triticum urartu* (NRT2.1, EMS65311; NRT2.4, EMS46096, NRT2.5, EMS50263); *Zea mays* (NRT2.1, XP_008645163; NRT2.2, NP_001105195; NRT2.3, XP_008656795; NRT2.7, AQK44570); *Hordeum vulgare* (NRT2.5, ABG20828; (NRT2.6, ABG20829); *Egeria densa* (NRT2, BAK51923); *Selaginella moellendorffii* (NRT2.1, XP_002993278; NRT2.4, XP_002966266); *Chlamydomonas reinhardtii* (NRT2.1, XP_001696789); *Hansenula polymorpha* (YNT1; CAA93631); *Aspergillus nidulans* (CRNA, XP_658612).

Table S1. List and description of primers used for cloning into pGEM®-T Easy and pDONR vectors**.** The restriction targets added to the gene-specific sequence are highlighted in gray. Oligonucleotide primer sequences containing attB1/attB2 Gateway® recombination sites are highlighted in bold (attB short sites) and underlined (attB adapter sites).

**Gene**		**Primer Sequence 5′ → 3′**	**Aplication**
NRT2	Fw attB1 short	**AAAAAGCAGGCT**ATGTCTGATCATGAGTTTGATG	Addition of attB short sites<br>PCR diagnostic
	Rv attB2 short	**AGAAAGCTGGGT**CTAAACAATGATGTTTGGGGAT	
	Fw attB1 short	GGGGACAAGTTTGTACA**AAAAAGCAGGCT**	Addition of attB adapter sites
	Rv attB2 short	GGGGACCACTTTGTACA**AGAAAGCTGGGT**	
NAR2	Fw attB1 short	**AAAAAAGCAGGCT**ATGTATTCTTCCTTCCTCTCC	Addition of attB short sites<br>PCR diagnostic
	Rv attB2 short	**AGAAAGCTGGGT**CTTTGTTCTTCCTTCTCCGGTC	
	Fw attB1 adapter	GGGGACAAGTTTGTACA**AAAAAGCAGGCT**	Addition of attB adapter sites
	Rv attB2 adapter	GGGGACCACTTTGTACA**AGAAAGCTGGGT**	
NRT2	Fw BamHI	GGATCCATGTCTGATCATGAGTTTGATGATC	cDNA amplification and restriction sites addition
	Rv XbaI	TCTAGACTAAACAATGATGTTTGGGGATTGG	
NAR2	Fw BamHI	GGATCCATGTATTCTTCCTTCCTCTCCG	cDNA amplification and restriction sites addition
	Rv XbaI	TCTAGATTATTTGTTCTTCCTTCTCCGGT	
